# Renal Cell Carcinoma Masquerading as Pyonephrosis — A Case Report of A Rare Presentation

**DOI:** 10.15586/jkc.v12i2.330

**Published:** 2025-04-03

**Authors:** Vishnu Pratap, Prakash Pawar, Ajit Sawant

**Affiliations:** Department of Urology, Lokmanya Tilak Municipal Medical College and General Hospital, Mumbai, Maharashtra, India

**Keywords:** Renal Cell Carcinoma, Pyonephrosis, Malignancy

## Abstract

We describe here an atypical case of pyonephrosis which on further evaluation turned out to be a renal cell carcinoma (RCC). The clinical presentation of the patient was that of an infective etiology. However, the renal mass associated with renal vein thrombus and lung metastasis was later diagnosed based on the clinical deterioration of the patient even after insertion of percutaneous nephrostomy. In this case, an underlying renal cancer was probably complicated secondarily leading to pyonephrosis which was the initial presenting manifestation which led to a delay in diagnosis. Pyonephrosis is usually associated with Xanthogranulomatous pyelonephritis. Association of RCC with pyonephrosis is extremely rare and hence seldom reported. Our patient later on underwent radical nephrectomy and the histopathology report was suggestive of RCC. We have described here the clinical manifestations and diagnostic issues of such a case. This case provides evidence that RCC should be kept as one of the differentials in such patients.

## Introduction

Pyonephrosis is infected hydronephrosis with suppurative destruction of renal parenchyma (1). Purulent exudate collects in the hydronephrotic collecting system and forms an abscess. If not recognized and treated promptly, this infectious process may progress, often resulting in clinical deterioration with urosepsis. The preoperative diagnosis of renal mass lesions has become much more accurate with modern imaging modalities like ultrasonography (US), computed tomography (CT), and magnetic resonance. Nevertheless, primary renal tumors have very rarely been reported to mimic pyonephrosis, renal abscess, or perinephric abscess (2).

## Case Report

A 46-year-old presented with pain in the left flank with fever and chills on and off since 15 days. He had generalized weakness for the past 1 week with a past history of open pyelolithotomy done 8 years back. The patient had tachycardia (100/min), pallor, and left flank tenderness with guarding. There was no palpable lump.

**Figure 1:**
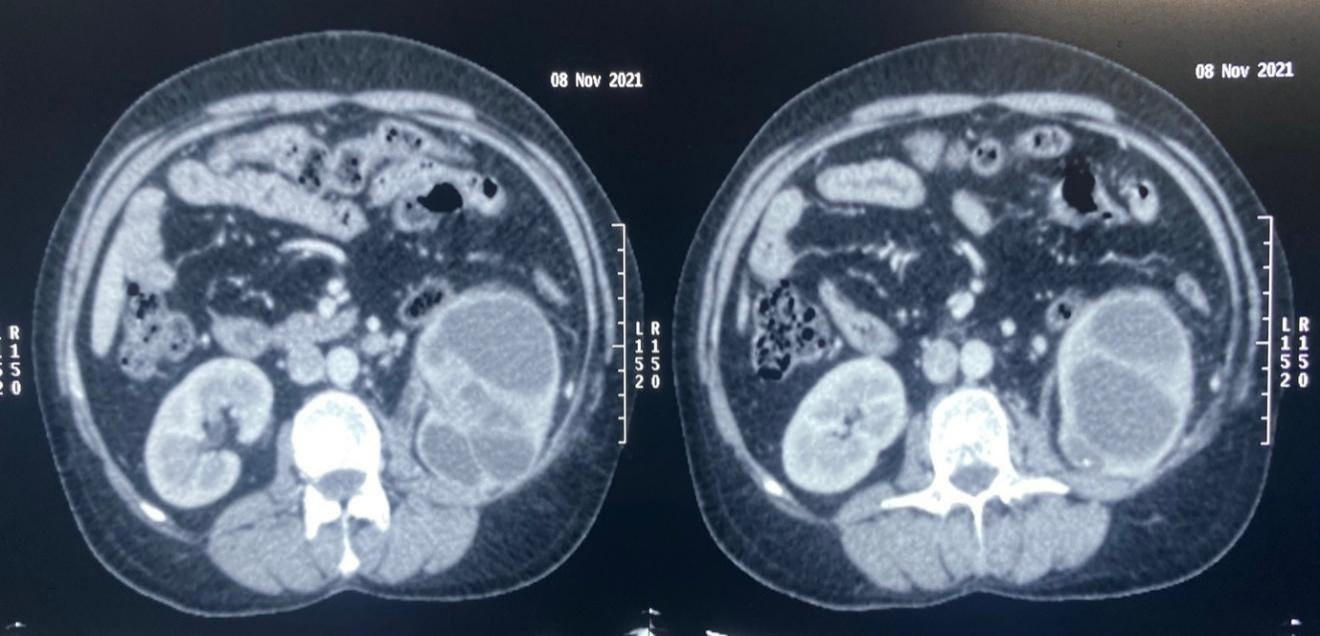
First computed tomography urography.

The patient was admitted, and third-generation cephalosporins were started. Blood culture and urine culture were sent. He had raised counts of 24,500/mm^3^ with a creatinine of 1.1. Ultrasound showed left-sided gross hydronephrosis with multiple echoes, and CT urography showed a left hydronephrotic kidney with no obvious obstructive calculus (Figure 1).

Left percutaneous nephrostomy (PCN) insertion was done, and 200 cc of frank pus was drained and sent for culture. Patient improved symptomatically, and the PCN output decreased to minimal after 2 days. Patients’ counts increased to 28500/mm^3^. Repeat ultrasound showed hydronephrosis with septations and echoes within PCN in situ. Hence, a second PCN was inserted in mid-pole, which drained 50 cc frank pus but eventually decreased to nil.

Patient had persistent fever spikes and raised counts even after PCN insertion and antibiotics. Hence, a CT chest was done to rule out any other source of infection.

**Figure 2:**
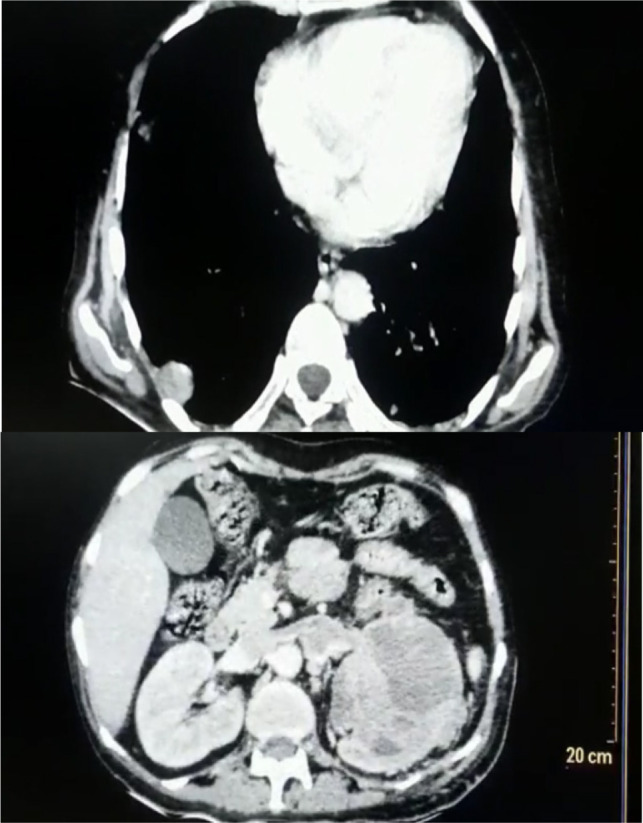
Computed tomography chest with abdomen.

**Figure 3:**
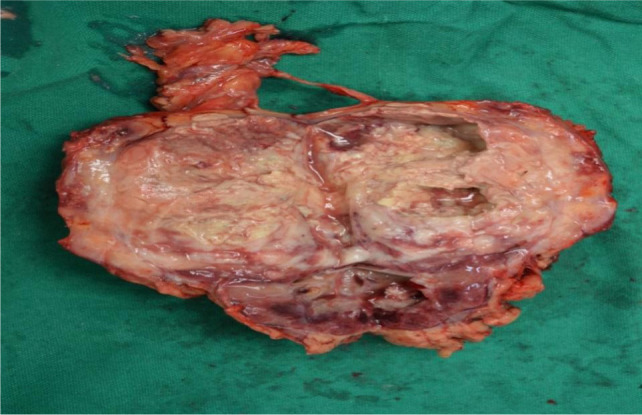
Postoperative specimen.

CT chest showed a pulmonary nodule on the right lung and left renal vein thrombus and a left renal mass, which was not seen on the previous CT scan (Figure 2). Decision for left radical nephrectomy was taken in view of persistent fever spikes with raised WBC counts. Open left radical nephrectomy via a thoracoabdominal approach was done. The left kidney along with the renal vein containing thrombus was removed (Figure 3).

**Figure 4:**
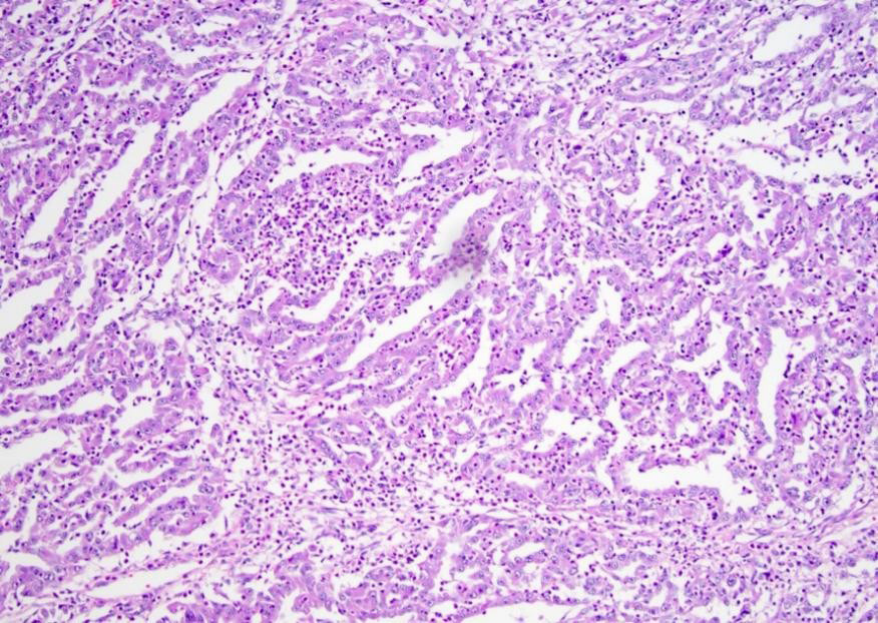
Histopathology image.

Postoperative course was uneventful except that the WBC counts were persistently raised over 20,000/mm^3^. The patient did not have any fever spikes postoperatively and was discharged on postoperative day 10.

Histopathology report was suggestive of high-grade collecting duct carcinoma (CDC) of size 10×7×6 cm with involvement of para-aortic lymph nodes and thrombus confined to the left renal vein. The patient succumbed to the disease within a month of surgery.

## Discussion

The case described here presented a diagnostic dilemma as its presentation pointed toward an infective etiology, which was not the case. A presentation with fever, leukocytosis, and presence of dense internal echoes on imaging puts an inflammatory pathology as the first diagnosis. Before the final diagnosis is made, the patient is usually subjected to multiple diagnostic tests, which leads to a delay in treatment which is what happened in the present case. Thus, while the diagnosis of pyonephrosis can be made with imaging, ascertaining the source of infection is often complex. This is of utmost significance to plan appropriate curative therapeutic strategies. Renal cell carcinoma (RCC) may rarely involve the calyces and pelvic infundibulae, resulting in extensive renal destruction with pyonephrosis.

Renal malignancies are uncommonly associated with inflammatory diseases such as renal abscess (3), pyelonephritis, xanthogranulomatous pyelonephritis (4), and rarely pyonephrosis (3), which can lead to a delay in the detection of cancer. The systemic signs of inflammation and malignancy can be mixed, and imaging studies sometimes cannot distinguish between these two diseases, leading to misinterpretation in standard imaging procedures (5). CT shows hydronephrosis and ultrasound shows dilatation with echoes within. However, because some infiltrative renal tumors may have an appearance similar to that of focal pyelonephritis, extension of the acute inflammatory process into the perirenal soft tissues may give the appearance of a renal malignancy. RCC on CT scan may be just a subtle lesion on the renal septae, and cysts and histopathological examination of the surgical specimen may be the only way to definitely rule out renal cancer (6). Sonography or CT findings of renal abscesses also reveal a well-defined heterogenous mass that at times may simulate a renal malignancy. In these conditions, renal malignancy has been confirmed after malignancy progression or metastasis. In some patients, there may be frank clinical features mimicking a renal abscess in an underlying tumor, leading to misdiagnosis and delay in treatment (7). Therefore, as the prognosis of renal malignancy is very different from pyelonephritis or pyonephrosis, the possibility of renal malignancy should always be kept in mind and followed up in an imaging study. On histopathology, papillary RCC presents with necrosis. This is also seen in tumors which are larger in size (8). This necrosis provides ground for the hematogenous bacteria to settle inside the tumor leading to abscess formation (9,10).

A brief outline of similar cases is shown below.

**Table:** 

Sr. No.	Case	Outcome
1.	Xanthogranulomatous pyelonephritis with associated RCC (11)	Radical nephrectomy with specimen histopathology of clear cell RCC
2.	Acute pyelonephritis including an overlooked RCC (12)	RCC was diagnosed 5 years after acute pyelonephritis for which appropriate treatment was done
3.	Clear cell type of renal cell carcinoma presenting with emphysematous pyelonephritis in young nondiabetic patient (13)	Radical nephrectomy in view of suspicious mass on imaging with histopathology findings of clear cell RCC

Amongst the cases described above, the first case showed imaging features suggestive of RCC. The second case developed RCC 5 years later. The last case, however, was not strongly suggestive of RCC on imaging but emphysematous pyelonephritis led to the plan for nephrectomy which later turned out to be RCC on histopathology.

Our case did not show features of RCC on imaging but showed pyonephrosis which led to percutaneous nephrostomy insertion, hence, the diagnostic dilemma. The RCC in our case was aggressive and quickly developed into renal vein thrombus which was made clear on histopathology which was suggestive of collecting duct RCC. These features were unique about our case if compared to the cases described above.

CDC is a rare and aggressive form of RCC arising from the principal cells of the collecting duct. One-third of the cases present with metastatic disease, but many present in a manner similar to conventional RCC or urothelial carcinoma (UC). Case reports have described CDC associated with deep vein thrombosis, extensive coagulative necrosis, or leukocytosis (14). However, these cases are atypical. Increased leucocytosis was seen in our case, which can be increased because of granulocyte-colony stimulating factor production. The prognosis of CDC is poor as it is an aggressive disease, and one-third of the cases are metastatic on presentation. Pepek et al. found that 3-year survival rates for localized, regional, and distant disease were 93%, 45%, and 6%, respectively (15). CDC is a pathological diagnosis. The diagnosis is made if the lesion shows desmoplastic reaction, infiltrative pattern, or high-grade features. Though several treatment options have been implemented with some success, a standard chemotherapy regimen is not yet established because of the rarity of CDC. Gemcitabine-cisplatin/carboplatin has proven to be beneficial in the treatment of CDC.

## Conclusion

In our case, the patient presented with all symptoms, signs, and imaging suggestive of an inflammatory pathology. Based on that, PCN insertion was done, but the patient did not improve and hence, further evaluation was done which revealed an RCC. Hence, clinicians should always include RCC as a differential in mind while evaluating such atypical cases to prevent delay in diagnosis and treatment. Careful interpretation of imaging and correlation with clinical features is of utmost importance to prevent a delay in diagnosis.
